# Disruption of prefrontal and superior temporal gyrus white matter networks in antipsychotic-treated schizophrenia with comorbid metabolic syndrome

**DOI:** 10.3389/fpsyt.2026.1878935

**Published:** 2026-08-28

**Authors:** Ying Li, Qianqian Liu, Xinyue Chen, Shiji Peng, Hangyu Li, Kaike Liao, Rui Yu, Nian Liu

**Affiliations:** 1 Department of Radiology, Affiliated Hospital of North Sichuan Medical College, Nanchong, China; 2 Nuclear Medicine and Radiation Safety Key Laboratory of Sichuan Province, North Sichuan Medical College, Nanchong, China

**Keywords:** diffusion tensor imaging, metabolic syndrome, schizophrenia, structural network, white matter

## Abstract

**Background:**

Metabolic syndrome (MS) is a frequent comorbidity in schizophrenia, yet the characteristics of white matter (WM) network alterations in this subgroup remain largely unknown. This study examined distinctive WM structural network disruptions in schizophrenia patients with comorbid MS and explored whether these alterations mediate the relationship between schizophrenia and metabolic abnormalities.

**Methods:**

Twenty-six schizophrenia patients with MS (SZ-MS), twenty-two without MS (SZ-nMS), and twenty-four age- and sex-matched healthy controls (HC) underwent diffusion tensor imaging. Individual WM structural networks were constructed, and group differences in connectivity and topological organization were analyzed using network-based statistics and graph theory, controlling for age and sex.

**Results:**

Both SZ-MS and SZ-nMS patients exhibited reduced FA-weighted connectivity in prefrontal, parietal, and temporal cortices, thalamus, and hippocampus compared to HC. Relative to SZ-nMS, the SZ-MS group exhibited significant reduction in prefrontal FA-weighted connectivity. Topological metrics revealed global and nodal disruptions in both patient groups, notably in prefrontal cortex, bilateral thalamus, supramarginal gyrus, left precuneus, and right superior temporal gyrus. The SZ-MS group demonstrated greater nodal efficiency impairments in prefrontal and right superior temporal gyrus regions. Further exploratory analyses revealed that WM network metrics correlated with triglycerides, BMI, waist circumference, hip circumference, and fasting glucose. Additionally, reduced nodal efficiency in the right superior temporal gyrus exhibited a potential mediating pathway between SZ-MS and elevated BMI.

**Conclusion:**

These findings indicate that SZ-MS patients display characteristic WM network disruptions, predominantly in prefrontal and right superior temporal areas. These alterations are associated with metabolic abnormalities, providing neuroimaging evidence that may clarify the neuropathological mechanisms connecting schizophrenia and metabolic dysfunction.

## 1 Introduction

As a chronic psychiatric disorder, schizophrenia is defined by profoundly disturbed cognition, emotion, and behavior, leading to substantial social and occupational dysfunction. Its high recurrence rate, associated disability, and suboptimal treatment adherence contribute to a considerable public health burden (1, 2). Antipsychotic medications, particularly second-generation agents, are the cornerstone of treatment and are generally effective for symptom control (3, 4). However, prolonged use is frequently accompanied by adverse metabolic changes, such as increased weight, dyslipidemia, and insulin resistance, which collectively elevate metabolic syndrome (MS) risk (5). The reported prevalence of MS among individuals with schizophrenia ranges from 40% to 43%, significantly exceeding that of the general population (6). MS aggravates psychiatric symptoms, impairs cognition, reduces medication compliance, and increases relapse risk, thereby contributing to poorer clinical outcomes and higher discontinuation rates (7–9). Despite these clinical implications, the neural correlates and imaging biomarkers underlying schizophrenia comorbid with MS remain poorly characterized.

Previous investigations have primarily focused on the systemic metabolic and physiological aspects of MS in schizophrenia, while neuroimaging evidence concerning brain structural and functional alterations is comparatively limited. Structural MRI studies have shown reductions in total brain volume, orbitofrontal cortical thickness, and reward circuitry volumes in schizophrenia patients with MS relative to those without MS (10). Functional MRI (fMRI) research has revealed abnormal functional coupling in the bilateral middle frontal gyrus (11) and reduced regional homogeneity in the inferior orbitofrontal cortex (12). Other findings include hypoperfusion in the orbitofrontal cortex and greater insula and middle/superior frontal gyri connectivity (13). Nevertheless, these studies primarily emphasize gray matter and functional activity, leaving the microstructural properties of white matter (WM) networks largely unexplored.

The WM connectome forms the anatomical backbone for efficient neural communication, and graph-theoretical approaches provide a powerful framework for quantifying its organization and efficiency (14, 15). Wu and colleagues first employed fiber number (FN)-based graph analysis to reveal disrupted topological architecture within the WM networks of schizophrenia patients with comorbid MS (16). Although informative, FN-based measures primarily reflect global tract count and are susceptible to biases related to tractography seeding and fiber length, which may limit their sensitivity to localized microstructural abnormalities. In contrast, network-based statistics (NBS) can identify specific connectivity subnetworks exhibiting significant alterations while effectively controlling the family-wise error rate (FWER) (17, 18), thereby complementing conventional graph-theoretical approaches. Furthermore, fractional anisotropy (FA), derived from diffusion tensor imaging (DTI), is sensitive to microstructural tissue properties such as myelination and axonal density (19, 20). Although FA is influenced by multiple biological and methodological factors and is not a direct or specific measure of axonal integrity, it provides a useful summary of WM microstructure along reconstructed pathways. Compared with FN-based measures, FA-weighted network analysis may offer a more refined characterization of microstructural alterations in SZ-MS patients. Therefore, integrating FA-weighted NBS with graph-theoretical metrics may provide complementary insights into the WM network disruptions associated with schizophrenia and metabolic comorbidity.

Building upon this foundation, the present study enrolled schizophrenia patients with MS (SZ-MS), schizophrenia patients without MS (SZ-nMS), and demographically matched healthy controls (HC). By integrating graph-theoretical analysis with NBS using FA-weighted WM connectomes, we aimed to (1) characterize WM network alterations unique to SZ-MS and (2) examine their role in mediating the relationship between schizophrenia and metabolic abnormalities. This study is expected to identify key neuroimaging markers of schizophrenia with comorbid metabolic syndrome.

## 2 Materials and methods

This research received approval from the Ethics Committee of the Affiliated Hospital of North Sichuan Medical College (approval number: 2022ER416-1) and adhered to the principles of the Declaration of Helsinki. All participants were fully informed of the study’s procedures, and written informed consent was obtained prior to participation.

### 2.1 Participants

From psychiatric hospitals in Guang’an City, Sichuan Province, China (specifically, Wusheng Bashu Psychiatric Hospital and Wusheng Ruikang Psychiatric Specialist Hospital), patients diagnosed with schizophrenia were enrolled. Participants received no financial compensation but were reimbursed a fixed travel allowance for their participation. All participants were receiving standard clinical care during hospitalization. Inclusion criteria for schizophrenia were based on the DSM-5 (21), and included: ① age ≥ 18 years; ② right-handedness; ③ ongoing treatment with second-generation antipsychotic medications; ④ absence of comorbid psychiatric disorders or substance dependence; ⑤schizophrenia onset ascertained from patient/family reports, medical records, and the Nottingham Onset Schedule (22).

MS was diagnosed in accordance with the race-adjusted criteria outlined by NCEP ATP-III (23). A diagnosis required a minimum of three of the following conditions: ① central obesity, representing a waist circumference ≥80 cm in women or ≥90 cm in men; ② reduced high-density lipoprotein cholesterol (HDL-C), defined as <1.29 mmol/L (50 mg/dL) in women or <1.03 mmol/L (40 mg/dL) in men, or pharmacological management for low HDL-C; ③ elevated triglycerides (TG) ≥1.7 mmol/L (150 mg/dL) or current treatment for hypertriglyceridemia; ④ hypertension, indicated by systolic/diastolic blood pressure ≥130/85 mmHg, or a prior diagnosis and ongoing treatment for hypertension; and ⑤ raised fasting blood glucose (FBG) ≥5.6 mmol/L (100 mg/dL) or previously diagnosed type 2 diabetes mellitus.

HC participants were recruited through community advertising and matched to the schizophrenia groups on sex, age, and education. All HCs were confirmed to be right-handed and denied any history of psychiatric or neurological disorders. As part of the screening process, the SCID-NP was administered with the dual purpose of confirming that participants themselves had no psychiatric illness and ensuring that none of them had a first-degree relative with a psychiatric diagnosis.

For both schizophrenia patients and HC, exclusion criteria were as follows: ① contraindications to MRI scanning (e.g., claustrophobia, metallic implants); ② history of alcohol or substance abuse; ③ previous organic brain lesions or significant head trauma; ④ severe systemic illnesses such as hepatic cirrhosis or cardiovascular disease; and ⑤ poor-quality DTI data or incomplete brain coverage.

Medical record reviews confirmed that all schizophrenia participants were free of MS prior to their most recent hospitalization. *A priori* sample size estimation was performed using G*Power 3.1.9.7 (24). Assuming an effect size of *f* = 0.40, a significance level (α = 0.05), and a desired power (1-β = 0.80) for a one-way analysis of variance with three groups, the required total sample size was calculated to be 66 participants. Ultimately, the final analysis comprised 72 participants, including 26 SZ-MS (13 males and 13 females; mean age 41.69 ± 10.34 years), 22 SZ-nMS (15 males and 7 females; mean age 38.18 ± 10.00 years), and 24 HC (9 males and 15 females; mean age 40.38 ± 9.77 years). Among SZ-MS patients, 2 received clozapine monotherapy, 9 were on risperidone monotherapy, and 15 received combination therapy; the SZ-nMS group comprised 3, 7, and 12 patients under the corresponding regimens. Daily antipsychotic dosages were standardized to chlorpromazine (CPZ) equivalents for comparison across medication types (22, 25).

### 2.2 Clinical data collection

Data collection for clinical, demographic, and metabolic data coincided with the MRI scan date. Demographic information included sex, age, height, weight, illness duration, and years of education. Metabolic parameters included waist circumference, blood pressure, body mass index (BMI), low-density lipoprotein cholesterol (LDL-C), HDL-C, total cholesterol (TC), TG, and FBG.

All clinical scales were administered by trained psychiatrists with at least five years of clinical experience, who underwent standardized training. Scoring and clinical interpretation were supervised by a researcher with neuropsychological qualifications. The Positive and Negative Syndrome Scale (PANSS) was used to evaluate psychiatric symptom severity, with higher scores indicating greater psychopathology (26). In healthy controls, the PANSS was administered solely to screen for and exclude the presence of psychotic-spectrum symptoms and to maintain procedural uniformity across all participants. Cognitive function was assessed using the Brief Assessment of Cognition in Schizophrenia (BACS), which evaluates six cognitive domains: working memory, verbal memory, verbal fluency, motor speed, attention, and executive functioning (27). Lower BACS scores reflect poorer cognitive performance. Adverse effects of treatment were assessed using the Treatment Emergent Symptom Scale (TESS) and the Rating Scale for Extrapyramidal Side Effects (RSESE), where higher scores indicate more severe side effects (28).

### 2.3 Image data acquisition

Imaging data were acquired using a 3.0 Tesla MRI system (Discovery 750, GE Healthcare) at the Affiliated Hospital of North Sichuan Medical College. Standardized instructions required participants to maintain stillness, keep their eyes closed, stay awake, and avoid focused mental activity throughout the scan. To mitigate motion artifacts and acoustic interference, foam padding and earplugs were utilized.

DTI data were acquired using a diffusion-weighted spin-echo echo-planar imaging sequence with the following parameters: repetition time (TR) = 7900 ms, echo time (TE) = 86 ms, flip angle = 90°, slice thickness = 2 mm, voxel size = 2 × 2 × 2 mm^3^, field of view (FOV) = 208 × 208 mm^2^, acquisition matrix = 104 × 104, and diffusion weighting (b-values) = 0 and 1000 s/mm^2^ with 30 non-collinear diffusion gradient directions. High-resolution T1-weighted anatomical images were obtained using a spoiled gradient-echo sequence with the following settings: TR = 7 ms, TE = 3 ms, flip angle = 9°, slice thickness = 1 mm, voxel size = 1 × 1 × 1 mm^3^, FOV = 256 × 256 mm^2^, and acquisition matrix = 256 × 256.

### 2.4 Image data processing and network construction

DTI preprocessing and network construction were undertaken using FSL (https://fsl.fmrib.ox.ac.uk/fsl) and the Pipeline for Analyzing Brain Connectivity Images (PANDA) toolbox. DTI and T1-weighted images were first converted from DICOM to NIFTI format. Head motion and eddy current distortions were corrected using FSL’s eddy tool, with all images aligned to the first b0 reference image. Susceptibility-induced distortions were corrected using FSL’s topup based on reverse-phase-encoded b0 images. Non-brain tissue was then removed and FA maps were computed. Quality control was performed at key preprocessing steps. After eddy correction, automated QC metrics were generated using FSL’s tool to assess residual motion and outlier slices. Additionally, all diffusion-weighted images were visually inspected for severe artifacts prior to tractography. Participants with mean frame-wise displacement greater than 0.3 mm or absolute rotation greater than 2° were excluded. Deterministic fiber tracking was conducted over the whole brain with the following parameters: FA threshold = 0.2, angular threshold = 45°, step length = 1 mm, and seed density = 1 seed per voxel. Tracking was initiated from all WM voxels with FA > 0.2 and terminated when FA dropped below 0.2 or when the turning angle exceeded 45°.

Node Definition: The brain was parcellated into 90 cortical and subcortical regions using the Automated Anatomical Labeling (AAL) atlas, with each region defined as a network node. Anatomical correspondence was achieved by spatially normalizing the T1-weighted images to the Montreal Neurological Institute (MNI) ICBM152 standard template. The transformation parameters derived from this step were subsequently utilized to align and parcellate each subject’s DTI data. The accuracy of spatial normalization was visually verified for each subject by overlaying the transformed T1-weighted images onto the MNI template. Subjects with obvious registration errors were excluded from further analysis.

Edge Definition: Edges between any two nodes were defined by the mean FA value of all fiber tracts connecting the respective regions. This edge weight serves as a streamline-derived summary of the underlying WM microstructure, specifically the mean diffusion anisotropy along reconstructed pathways. It is not a direct measure of anatomical connection strength or tract count. FA is sensitive to microstructural tissue properties, such as myelination and axonal density. This sensitivity allows FA-based measures to complement fiber count-based metrics. Fiber count-based metrics are more susceptible to biases related to tractography seeding and fiber length. For any pair of brain regions, we identified all fiber tracts with one endpoint in each region, calculated the mean FA value across these tracts, and defined this mean as the edge weight between the two regions, thereby generating a symmetric 90 × 90 FA-weighted adjacency matrix for each participant.

### 2.5 White matter network analysis

Because there is no universally accepted thresholding approach for structural networks, sparsity thresholds were applied to ensure network stability and comparability across participants. A network was retained if it exhibited a mean degree greater than log(N/2) and a small-worldness index exceeding 1.1 (29, 30). Sparsity thresholds ranged from 0.10 to 0.40 (in 0.01 increments) to assess consistency and mitigate spurious connections. For each global and nodal metric, the area under the curve across the sparsity range was computed for each participant.

Global and regional topological metrics of the WM network were computed using the GRETNA toolbox (https://github.com/sandywang/GRETNA). Global metrics included normalized clustering coefficient (Gamma), characteristic path length (Lp), normalized characteristic path length (Lambda), small-worldness (Sigma), clustering coefficient (Cp), global efficiency (Eg), and local efficiency (Eloc). Cp and Gamma reflect local segregation. Lp and Lambda reflect global integration. Sigma captures the balance between segregation and integration. Eg and Eloc quantify information transfer efficiency at the whole-network and regional levels, respectively. Nodal metrics encompassed nodal degree centrality (DC), nodal efficiency (NE), and nodal shortest path length (NLP). DC represents the number of direct links to a node, reflecting its centrality within the network. NE quantifies the efficiency of information exchange between nodes, whereas NLP characterizes the extent to which a node facilitates network-wide integration (15).

### 2.6 Statistical analysis

Data are shown as means ± standard deviation or median (interquartile range), depending on distributional characteristics. Data normality and variance homogeneity were assessed using the Shapiro-Wilk and Levene tests, respectively. For variables following a normal distribution, between-group differences were examined using independent-samples t-tests (two groups) or one-way analyses of variance with *post hoc* comparisons (three groups). Skewed data were assessed using Mann-Whitney U tests (two groups) or Kruskal-Wallis tests (three groups) as appropriate. Group differences in sex distribution were assessed with the chi-square test. To minimize the influence of potential confounders, age and sex were included as covariates in all relevant analyses. To assess the robustness of our findings, we performed sensitivity analyses by additionally including years of education as a covariate in the same statistical models. The results remained essentially unchanged, with all significant group differences retaining the same direction and significance (**Supplementary Tables S1**–**S5**).

#### 2.6.1 White matter FA-weighted connectivity

NBS were applied to examine group differences in FA-weighted connectivity between network nodes (18, 31). Initially, two-sample t-tests were performed on each edge of the intergroup brain networks to generate a t-value map. An edge-level threshold of p < 0.01 was used to identify candidate connections. The significance of any interconnected suprathreshold components was then assessed via permutation testing (5,000 iterations), specifically by determining how often the size of the largest component in permuted data exceeded that in the actual data. FWER correction was applied, and subnetworks with corrected p < 0.05 were considered significant.

#### 2.6.2 Network topological properties

Analysis of covariance (ANCOVA) was conducted to assess group differences in both global and nodal network measures across the SZ-MS, SZ-nMS, and HC groups, controlling for covariates. Bonferroni correction was applied to adjust for multiple comparisons of nodal metrics. *Post hoc* pairwise contrasts were subsequently performed via Fisher’s least significant difference (LSD) test.

#### 2.6.3 Correlation analysis

Within the patient cohort, Pearson correlation coefficients (normally distributed) or Spearman rank correlations (skewed) were computed to explore the associations between altered FA-weighted connectivity or network topology metrics and metabolic indicators of MS. Bonferroni correction was applied for multiple comparisons (α = 0.05/20/10), with a corrected p value < 0.00025 considered statistically significant.

#### 2.6.4 Mediation model analysis

Mediation analysis was performed using the SPSS PROCESS macro (v 4.2) (32). The independent variable (X) represented MS comorbidity status (SZ-MS vs. SZ-nMS), the mediator (M) corresponded to WM network nodes or connections exhibiting significant group differences, and the dependent variable (Y) was the metabolic parameter that differed significantly between groups. The significance of the mediation (indirect) effect was established through a nonparametric bootstrap procedure (5,000 resamples), which produced 95% bias-corrected confidence intervals. The statistical significance of the mediation effect was determined by whether the bootstrap confidence interval excluded zero.

## 3 Results

### 3.1 Participant characteristics

The demographic and clinical characteristics of the study sample are presented in **Table 1**. No significant group differences were observed in age, sex, education, LDL-C, or HDL-C among the SZ-MS, SZ-nMS, and HC groups. Likewise, the two groups did not differ significantly in terms of illness duration, CPZ equivalent, TESS, or RSESE scores (p > 0.05).

**Table 1:** Demographic information and clinical characteristics of the participants.

Characteristics	SZ-MS group (n=26)	SZ-nMS group (n=22)	HC group (n=24)	Test statistic (*F/t/U/H/χ²*)	*p* value	*Post hoc* analysis		
						SZ-MS vs. SZ-nMS	SZ-MS vs. HC	SZ-nMS vs. HC
Male/female	13/13	15/7	9/15	4.357	0.113	—	—	—
Age (years)	41.69 ± 10.34	38.18 ± 10.00	40.38 ± 9.77	0.733	0.484	—	—	—
Education (years)	7 (6, 9)	9 (6, 10)	9 (7.50, 11.50)	3.150	0.207	—	—	—
Illness duration (years)	9.81 ± 5.86	11.68 ± 6.62	—	-1.039	0.304	—	—	—
CPZ equivalent (mg/day)	219.55 (187.50, 409.37)	387.5 (228.12, 475)	—	207.000	0.100	—	—	—
BMI (kg/m^2^)	25.24 ± 2.85	20.89 ± 2.47	22.23 ± 2.75	16.501	<0.001^***^	<0.001^***^	<0.001^***^	0.292
LDL-C (mmol/L)	3.01 ± 0.75	2.69 ± 0.55	2.55 ± 0.87	2.682	0.076	—	—	—
HDL-C (mmol/L)	1.26 ± 0.57	1.57 ± 0.55	1.52 (0.90, 3.16)	4.834	0.089	—	—	—
TC (mmol/L)	4.68 ± 1.04	3.48 (2.91, 4.63)	4.45 (2.00, 5.06)	8.231	0.016^*^	0.019^*^	0.120	1.000
TG (mmol/L)	2.19 (1.91, 2.61)	1.58 (1.32, 2.01)	1.55 (1.38, 2.39)	13.572	0.001^**^	0.002^**^	0.013^*^	1.000
FBG (mmol/L)	5.80 (5.07, 6.45)	4.85 (4.37, 5.40)	—	151.500	0.005^**^	—	—	—
Waist circumference (cm)	91.01 ± 5.27	78.72 ± 6.79	—	7.056	<0.001^***^	—	—	—
Hip circumference (cm)	96.44 ± 3.80	89.81 ± 4.91	—	5.260	<0.001^***^	—	—	—
Systolic blood pressure	125.31 ± 18.49	111.95 ± 11.57	—	2.933	0.005^**^	—	—	—
Diastolic blood pressure	82.81 ± 11.33	75.55 ± 8.78	—	2.445	0.018^*^	—	—	—
PANSS total score	53 ± 12.42	55.82 ± 14.6	34 (33, 35)	47.561	<0.001^***^	1.000	<0.001^***^	<0.001^***^
RSESE	0.5 (0, 5)	0 (0, 4)	—	202.500	0.081	—	—	—
TESS	4 (2.75, 9.25)	2.5 (1.5, 7.5)	—	246.000	0.361	—	—	—
BACS total score	136.12 ± 53.06	120.86 ± 53.59	237.08 ± 45.15	36.757	<0.001^***^	0.909	<0.001^***^	<0.001^***^
Working memory	14 ± 8.25	10 (3, 17)	25 (23.25, 27)	33.887	<0.001^***^	0.558	<0.001^***^	<0.001^***^
Verbal memory	18.5 ± 9.45	14.14 ± 9.42	40.54 ± 13.47	39.493	<0.001^***^	0.520	<0.001^***^	<0.001^***^
Verbal fluency	17.42 ± 8.02	14.45 ± 7.01	27.54 ± 8.01	18.535	<0.001^***^	0.567	<0.001^***^	<0.001^***^
Movement speed	66 (37, 76)	67 (43, 74.5)	82 (78, 88)	20.392	<0.001^***^	1.000	<0.001^***^	<0.001^***^
Executive function	9.5 ± 6.77	8.45 ± 6.05	19 (15, 20.75)	24.752	<0.001^***^	1.000	<0.001^***^	<0.001^***^
Attention	19 (7.5, 34)	18.5 (3.75, 25.5)	46.21 ± 19.78	27.473	<0.001^***^	1.000	<0.001^***^	<0.001^***^

“—” not applicable; *p < 0.05, **p < 0.01, ***p < 0.001.

Data are presented as mean ± SD for normally distributed variables or median (interquartile range) for skewed variables. Statistical tests performed: group differences in age, BMI, LDL-C, BACS total score, verbal memory, and verbal fluency, one-way ANOVA test and *post hoc* test; gender, chi-square test; education, PANSS total score, HDL-C, TC, TG, working memory, attention, executive function, and movement speed, Kruskal-Wallis test and *post hoc* test; illness duration, waist circumference, hip circumference, systolic blood pressure, and diastolic blood pressure, independent sample t-test; chlorpromazine equivalent, TESS, RSESE, and FBG, Mann-Whitney U test.

Both SZ-MS and SZ-nMS patients exhibited significantly lower total BACS and higher PANSS scores compared with the HC group (p < 0.001), with no difference between the two patient groups. BMI and TG levels were significantly elevated in the SZ-MS group relative to both HC and SZ-nMS participants (p < 0.05). Additionally, TC was significantly higher in SZ-MS group compared with SZ-nMS group (p < 0.05). The SZ-MS group also demonstrated greater FBG, blood pressure, waist circumference, and hip circumference than the SZ-nMS group (p < 0.05).

### 3.2 White matter FA-weighted connectivity

**Figure 1:**
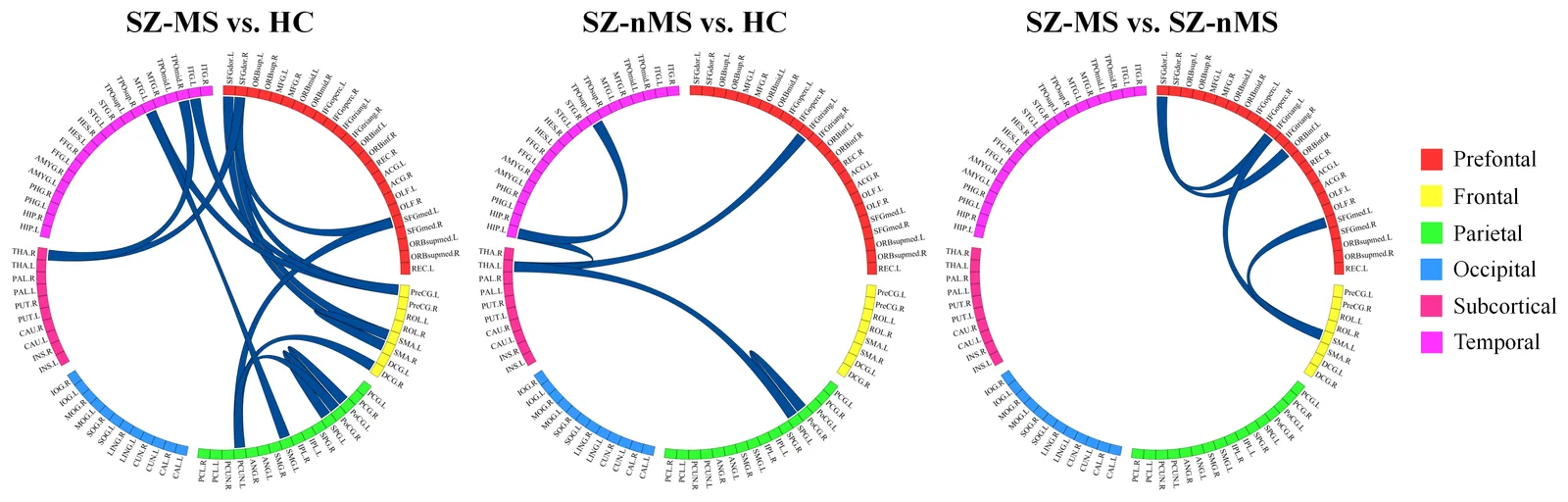
Differences in white matter FA-weighted connectivity among the SZ-MS, SZ-nMS, and HC groups. The blue curves indicate the lower FA values between the two regions. HC, healthy controls; SZ-MS, schizophrenia patients with comorbid metabolic syndrome; SZ-nMS, schizophrenia patients without metabolic syndrome.

NBS analysis revealed distinct patterns of reduced FA-weighted connectivity among three groups (**Figure 1**). Compared with HC, the SZ-MS group exhibited significantly decreased FA-weighted connectivity encompassing 16 nodes and 15 edges (p < 0.001), spanning both cortical and subcortical (thalamic) regions. The SZ-nMS group also showed a diminished subnetwork relative to HC, involving six nodes and five edges (p < 0.05), primarily located in the right postcentral gyrus, left inferior frontal gyrus (triangular part), left superior parietal gyrus, left hippocampus, left thalamus, and left superior temporal gyrus. Compared with SZ-nMS patients, the SZ-MS group demonstrated further reductions in FA-weighted connectivity comprising five nodes and four edges (p < 0.05). These alterations predominantly involved the left dorsolateral superior frontal gyrus, left inferior frontal gyrus (triangular and orbital parts), left medial superior frontal gyrus, and left supplementary motor area.

### 3.3 Global topological properties

**Figure 2:**
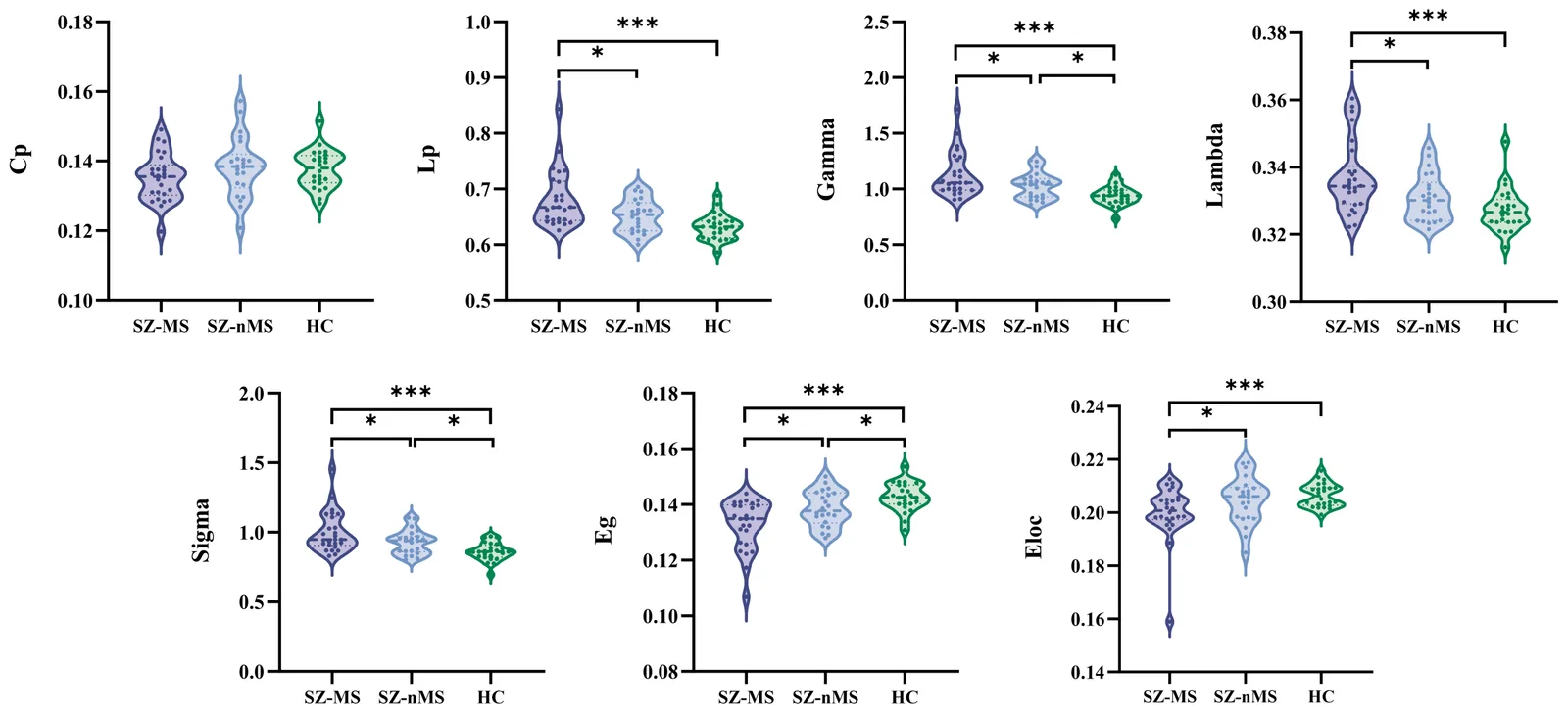
Differences in global topological properties among the SZ-MS, SZ-nMS, and HC groups. *p < 0.05, ***p < 0.001. HC, healthy controls; SZ-MS, schizophrenia patients with comorbid metabolic syndrome; SZ-nMS, schizophrenia patients without metabolic syndrome; Gamma, normalized clustering coefficient; Lp, characteristic path length; Lambda, normalized characteristic path length; Sigma, small-worldness; Cp, clustering coefficient; Eg, global efficiency; Eloc, local efficiency.

ANCOVA results indicated significant group differences in global network topology (**Figure 2**). *Post hoc* analyses revealed that, relative to HC, both schizophrenia groups displayed increased Gamma and Sigma values but significantly decreased Eg (p < 0.05). Moreover, the SZ-MS group demonstrated elevated Lp and Lambda as well as reduced Eloc compared with HC (p < 0.001 and p < 0.05, respectively). A comparison of the two patient groups indicated that SZ-MS individuals exhibited higher Lp, Gamma, Lambda, and Sigma, along with lower Eg and Eloc than SZ-nMS patients (p < 0.05).

### 3.4 Topological properties of nodes

**Figure 3:**
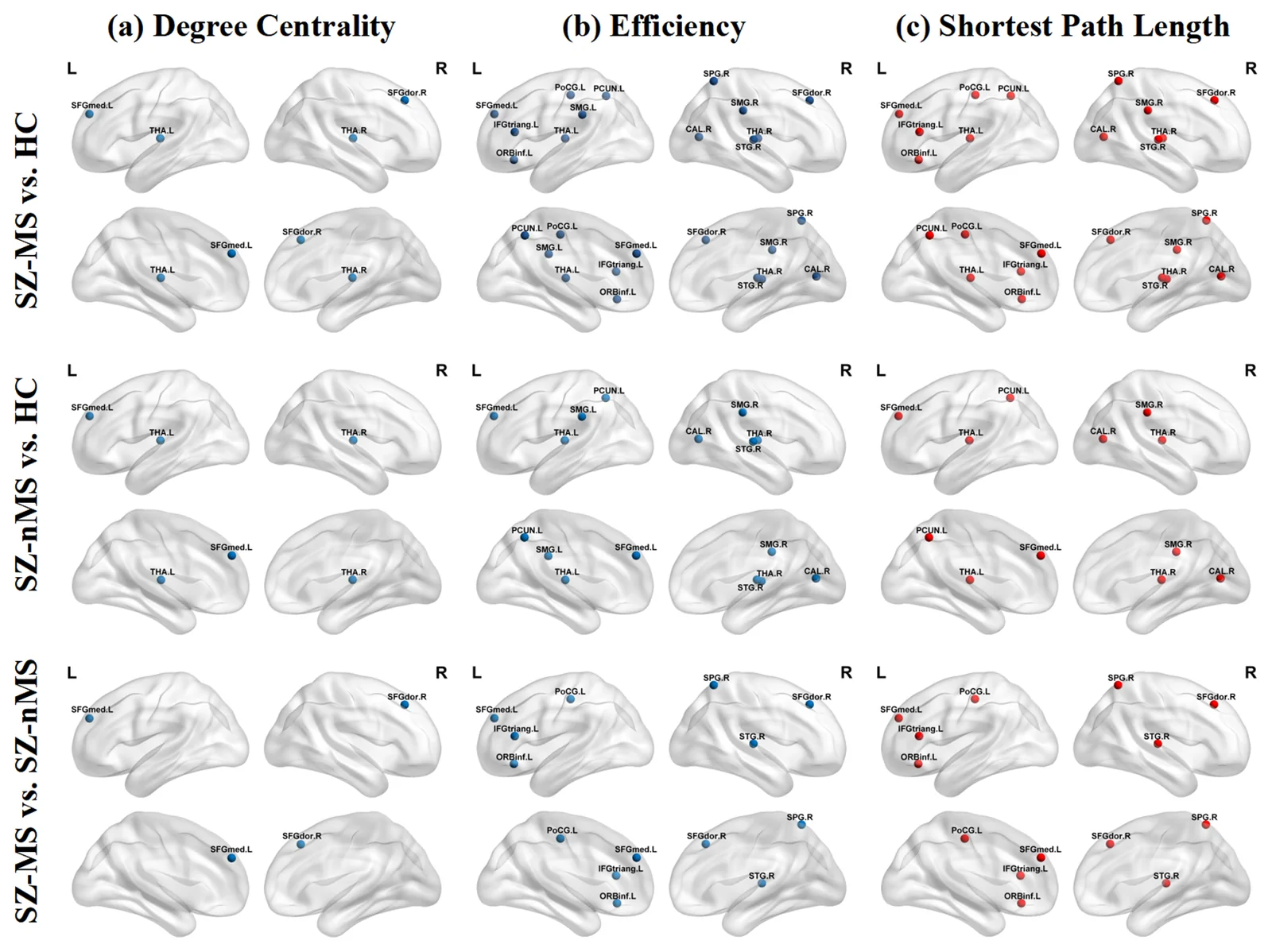
Differences in nodal network metrics **(a)** degree centrality, **(b)** efficiency, and **(c)** shortest path length among the SZ-MS, SZ-nMS, and HC groups. HC, healthy controls; SZ-MS, schizophrenia patients with comorbid metabolic syndrome; SZ-nMS, schizophrenia patients without metabolic syndrome; R, right; L, left; IFGtriang, the triangular part of the inferior frontal gyrus; ORBinf, the orbital inferior frontal gyrus; SFGdor, the dorsolateral superior frontal gyrus; SFGmed, the medial superior frontal gyrus; CAL, the calcarine fissure and surrounding cortex; PoCG, the postcentral gyrus; SPG, the superior parietal gyrus; SMG, the supramarginal gyrus; PCUN, the precuneus; THA, the thalamus; STG, the superior temporal gyrus.

ANCOVA with Bonferroni correction revealed significant group differences in nodal-level metrics across multiple regions (**Figure 3**). *Post hoc* analyses showed that, compared with HC, both schizophrenia groups exhibited reduced DC and NE, along with increased NLP in several areas including the supramarginal gyrus, left medial superior frontal gyrus, left precuneus, right calcarine fissure, right superior temporal gyrus, and bilateral thalamus (p < 0.05). In addition, SZ-MS patients demonstrated further nodal disruption in the prefrontal cortex, left postcentral gyrus, and right superior parietal gyrus (p < 0.05). Relative to SZ-nMS patients, the SZ-MS group exhibited more pronounced nodal impairments primarily in the left inferior frontal gyrus (triangular and orbital parts), left medial superior frontal gyrus, right dorsolateral superior frontal gyrus, right superior temporal gyrus, right superior parietal gyrus, and postcentral gyrus (p < 0.05).

### 3.5 Correlation between white matter network alterations and MS-related indicators

In the uncorrected analyses, several significant associations were observed between altered WM metrics and metabolic parameters within the schizophrenia cohort (**Table 2**). TG levels were inversely correlated with FA-weighted connectivity between the left dorsolateral superior frontal gyrus and the left inferior frontal gyrus (triangular part) (r = −0.395). Reduced NE in the right dorsolateral superior frontal gyrus, left inferior frontal gyrus (triangular part), and right superior temporal gyrus correlated negatively with BMI (r = −0.311, −0.330, and −0.435, respectively) and waist circumference (r = −0.361, −0.337, and −0.372, respectively). Decreased NE in the left inferior frontal gyrus (triangular part) also correlated with hip circumference (r = −0.381), while reduced NE in the left medial superior frontal gyrus correlated with FBG (r = −0.351). Conversely, increased NLP in the right dorsolateral superior frontal gyrus, left inferior frontal gyrus (triangular part), and right superior temporal gyrus showed positive correlations with BMI (r = 0.332, 0.349, 0.424, respectively) and waist circumference (r = 0.357, 0.369, 0.476, respectively). Elevated NLP in the left inferior frontal gyrus (triangular part) was also associated with hip circumference (r = 0.380), and higher NLP in the left medial superior frontal gyrus correlated positively with FBG (r = 0.351). No other significant associations between metabolic variables and brain regions were detected at the uncorrected level. However, after applying Bonferroni correction for multiple comparisons, none of these associations remained statistically significant (all corrected p > 0.05).

**Table 2:** Correlations between alterations in white matter networks and TG, BMI, waist circumference, hip circumference, or FBG.

Network metrics	TG		BMI		Waist circumference		Hip circumference		FBG	
	*r* value	*p* value	*r* value	*p* value	*r* value	*p* value	*r* value	*p* value	*r* value	*p* value
FA-weighted connectivity										
SFGdor.L - IFGtriang.L	-0.395	0.005^**^	—	—	—	—	—	—	—	—
Nodal efficiency										
IFGtriang.L	—	—	-0.330	0.005^**^	-0.337	0.019^*^	-0.381	0.008^**^	—	—
SFGdor.R	—	—	-0.311	0.032^*^	-0.361	0.012^*^	—	—	—	—
STG.R	—	—	-0.435	0.002^**^	-0.372	0.009^**^	—	—	—	—
SFGmed.L	—	—	—	—	—	—	—	—	-0.351	0.014^*^
Nodal shortest path length										
IFGtriang.L	—	—	0.349	0.015^*^	0.369	0.010^**^	0.380	0.008^**^	—	—
SFGdor.R	—	—	0.332	0.021^*^	0.357	0.013^*^	—	—	—	—
STG.R	—	—	0.424	0.003^**^	0.476	0.001^**^	—	—	—	—
SFGmed.L	—	—	—	—	—	—	—	—	0.351	0.014^*^

“—” not applicable; *p < 0.05, **p < 0.01.

R, right; L, left; IFGtriang, the triangular part of the inferior frontal gyrus; SFGdor, the dorsolateral superior frontal gyrus; STG, the superior temporal gyrus; SFGmed, the medial superior frontal gyrus.

### 3.6 White matter networks as mediators in SZ-MS and metabolic abnormalities

**Figure 4:**
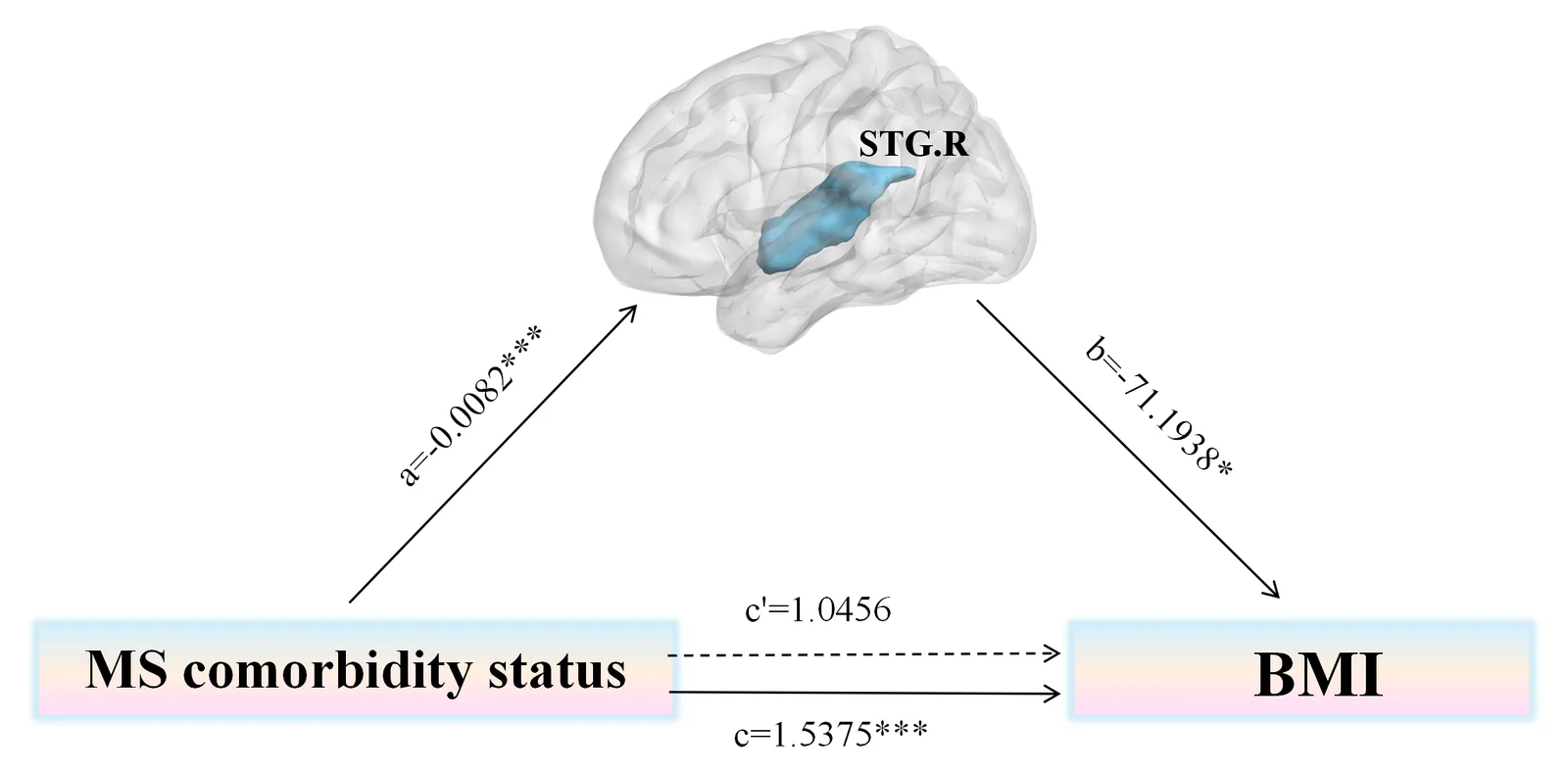
Exploratory mediation pathway of node efficiency in the right superior temporal gyrus linking metabolic syndrome comorbidity status to BMI. *p < 0.05, ***p < 0.001. c, total effect of MS comorbidity status (SZ-MS vs. SZ-nMS) on BMI; c’, direct effect of MS comorbidity status on BMI after introducing the mediator; a, effect of MS comorbidity status on nodal efficiency of the right superior temporal gyrus; b, effect of nodal efficiency in the right superior temporal gyrus on BMI; STG.R, the right superior temporal gyrus; MS, metabolic syndrome.

Mediation analysis revealed that NE in the right superior temporal gyrus showed a significant indirect effect in the relationship between SZ-MS and BMI (**Figure 4**). The total effect of MS comorbidity status (SZ-MS vs. SZ-nMS) on BMI was significant (β = 1.537, p < 0.001). After introducing right superior temporal gyrus NE as a mediator, the direct effect of MS comorbidity status on BMI became non-significant (β = 1.045, p = 0.100), whereas the indirect pathway via NE remained statistically significant (β = 0.581, BootSE = 0.293, 95% BootCI [0.025, 1.184]). When the same mediation analysis was performed with other significantly different brain regions as mediators, no statistically significant indirect effects were observed.

## 4 Discussion

This study integrated NBS and graph-theoretical analyses to examine WM structural network alterations in SZ-MS patients after antipsychotic exposure, as well as to explore the neural mechanisms linking schizophrenia with metabolic dysfunction. At the edge level, both schizophrenia groups exhibited diffuse reductions in FA-weighted connectivity relative to HC. Compared with the SZ-nMS subgroup, patients with SZ-MS displayed additional reductions in prefrontal FA-weighted connectivity. At the topological level, SZ-MS patients demonstrated more extensive alterations in nodal metrics, particularly within the prefrontal cortex and right superior temporal gyrus. These WM disruptions correlated with TG, waist circumference, hip circumference, BMI, and FBG. In addition, exploratory mediation analysis indicated that reduced NE in the right superior temporal gyrus was statistically consistent with a mediating role between SZ-MS and BMI. By employing FA-weighted edge analysis, these findings further extend the prior FN-based graph-theoretical study. Collectively, they provide complementary neuroimaging evidence for WM network alterations in SZ-MS and offer new insights into the neuroimaging mechanisms underlying the comorbidity of schizophrenia and metabolic abnormalities.

At the connectivity level, both schizophrenia groups showed markedly reduced FA-weighted connectivity linking the prefrontal, parietal, temporal, and thalamic regions, as well as connections between the parietal cortex and hippocampus, when compared with HC. This pattern is consistent with prior work showing widespread WM microstructural deficits in schizophrenia (33–35), which are believed to underlie impairments in higher cognitive and affective functions (34, 36, 37). Notably, compared with SZ-nMS patients, the SZ-MS group showed further reductions in connectivity involving the prefrontal cortex and supplementary motor area, which are key components of the default mode network and frontoparietal control network. These systems are essential for decision-making, executive control, and cognitive flexibility (38–40). Functional MRI studies have reported similar connectivity disruptions within these networks in patients with MS (41, 42). Moreover, individuals with abdominal obesity demonstrate impaired frontoparietal connectivity (43) and reduced coupling in the supplementary motor area (44). Because abdominal obesity is a major contributor to the onset and progression of MS (45, 46), these findings suggest that metabolic disturbances may exacerbate schizophrenia-related brain network disorganization by further compromising prefrontal WM microstructural integrity.

At the global network level, schizophrenia patients exhibited decreased integration and efficiency of WM networks relative to controls, a finding consistent with prior reports (16, 47, 48). The brain’s topological organization reflects its capacity for both local specialization and global information integration (49, 50). Interestingly, the SZ-MS group displayed significantly higher clustering coefficients and small-worldness indices than both SZ-nMS and HC groups, indicating an excessively clustered yet inefficient network organization. This pattern could reflect the impact of MS-related chronic metabolic stress, such as systemic inflammation and oxidative damage, impairs WM networks, ultimately impairing global communication efficiency (51, 52). Consequently, information transmission across distributed brain regions may be less efficient in SZ-MS patients.

At the nodal level, compared to the SZ-nMS group, the SZ-MS group demonstrated lower DC and NE, and higher NLP values within the dorsolateral and medial superior frontal gyri, as well as the left inferior frontal gyrus (triangular and orbital parts). The prefrontal cortex serves as a central hub for executive control, impulse regulation, and decision-making (53, 54). Damage in these regions may lead to disinhibited eating behavior and a preference for high-calorie foods, promoting obesity, insulin resistance, and, ultimately, the development of MS (55, 56). Wu and colleagues previously reported alterations in the medial prefrontal cortex of SZ-MS patients (16). In contrast, our study mainly observed nodal disruptions in the dorsolateral and medial superior frontal gyri, as well as in left inferior frontal gyrus (triangular part). This discrepancy may reflect methodological differences, as FN reflects fiber tract quantity, whereas FA is more sensitive to microstructural tissue properties. Nonetheless, both studies suggest that prefrontal network topological disruptions may play an important role in schizophrenia with comorbid MS. Supporting this, de Nijs et al. reported reduced gray matter volumes in reward-related circuits encompassing the orbitofrontal and medial prefrontal cortices (10), while Chen et al. observed decreased regional homogeneity in the inferior orbitofrontal gyrus in SZ-MS relative to SZ-nMS patients (12). Together, these findings indicate that both structural and functional disturbances within prefrontal regions, which are key nodes for reward processing and self-regulation, may constitute a shared neuropathological substrate linking schizophrenia and metabolic dysregulation.

In uncorrected exploratory analyses, elevated TG levels were linked to diminished FA-weighted connectivity between the dorsolateral superior frontal gyrus and the inferior frontal gyrus (triangular part) in the patient cohort. High TG levels have been shown to induce endothelial dysfunction in patients with MS (57), a process closely associated with cerebral tissue injury. Previous research has reported that schizophrenia patients with hypertriglyceridemia or central obesity exhibit abnormal WM microstructure (58). Consistent with these findings, our results suggest that elevated TG in SZ-MS patients may be associated with microstructural alterations in prefrontal circuits involved in executive function. Moreover, disruptions within prefrontal and right superior temporal gyrus nodes were correlated with higher BMI, larger waist and hip circumferences, and increased fasting blood glucose levels. These regions represent critical hubs within the neural networks supporting higher-order cognitive processes (59, 60). It is plausible that these metabolic disturbances may affect information processing efficiency in these regions, thereby influencing the regulation of eating behavior in schizophrenia patients.

A notable exploratory finding of this study is that node efficiency in the right superior temporal gyrus was statistically consistent with a mediating role in the relationship between SZ-MS and increased BMI. However, given the cross-sectional design, this mediation analysis does not demonstrate temporal precedence or causality. It remains equally plausible that metabolic changes influence WM microstructure, or that antipsychotic exposure drives both phenomena. Therefore, this finding should be interpreted as hypothesis-generating rather than as evidence of a mechanistic pathway. Although this region is classically associated with auditory perception and language comprehension (59, 61), accumulating evidence indicates its involvement in broader neural circuits related to obesity (62). Through its anatomical and functional connections with the insula, prefrontal cortex, and hypothalamus, the superior temporal gyrus contributes to the interoceptive perception of hunger and satiety (63, 64). Consequently, reduced NE in this area may reflect network dysfunction that disrupts energy homeostasis and metabolic regulation. Interestingly, while the prefrontal WM network exhibited the most pronounced alterations in the SZ-MS group, mediation analysis did not identify it as a significant intermediary between schizophrenia and metabolic indices. This suggests that the prefrontal and superior temporal networks may modulate metabolic function through distinct neurobiological mechanisms. The reasons underlying this dissociation remain uncertain and represent an important direction for future research.

This research is subject to several limitations. First, it was a preliminary exploratory investigation with a relatively small sample size. Although *a priori* sample size estimation was performed based on the effect size, the relatively small sample size may limit statistical power and estimation stability for graph-theoretical and mediation analyses. Replication in larger, multicenter, longitudinal cohorts is warranted. Second, although metabolic indicators were systematically assessed in schizophrenia patients, the influence of antipsychotic type, dosage, and treatment duration on brain structural networks could not be fully controlled. The present study primarily included patients receiving clozapine or risperidone, which reduces variability due to mixed pharmacotherapy but precludes conclusions regarding the effects of other antipsychotic agents. In addition, only partial lipid profiles were collected in the HC group, and comprehensive metabolic parameters were not assessed. Therefore, direct comparisons of metabolic profiles between the HC and patient groups were not feasible. Future studies should systematically collect complete metabolic data in control groups to more comprehensively control for confounding factors affecting WM networks. Third, given the cross-sectional design, the causal relationships between MS and WM alterations cannot be determined. Therefore, the mediating effects reported herein should be interpreted as exploratory findings rather than evidence of causal mechanisms. Furthermore, the cross-sectional design also limits the ability to track dynamic changes in metabolic parameters and brain network metrics during antipsychotic treatment. Future studies combining longitudinal follow-up with causal mediation analysis are warranted to further elucidate the temporal relationships and underlying mechanisms linking metabolic abnormalities to brain structural and functional impairments in schizophrenia patients. Fourth, Bonferroni correction was applied to the correlation analyses to control for multiple comparisons. However, none of the associations remained statistically significant after correction. Therefore, these correlation results should be interpreted as exploratory findings. Fifth, our DTI protocol used a single-shell acquisition with only 30 diffusion-encoding directions, which offers limited sensitivity to crossing fibers. This methodological constraint may affect tractography performance in regions with complex WM architecture. Future studies employing multi-shell acquisitions with higher angular resolution or probabilistic tractography would provide more robust characterization of white matter network organization.

## 5 Conclusion

In summary, this integrated analysis using network-based statistics and graph-theoretical modeling identified characteristic disruptions in both FA-weighted connectivity and network topology of WM in schizophrenia patients with metabolic syndrome. These abnormalities predominantly involve the prefrontal cortex and right superior temporal gyrus and are associated with metabolic dysfunction. The findings provide neuroimaging evidence that may elucidate the neurobiological mechanisms linking schizophrenia and metabolic syndrome.

## Data Availability

The raw data supporting the conclusions of this article will be made available by the authors, without undue reservation.

## References

[1] Jauhar S Johnstone M McKenna PJ . Schizophrenia. Lancet. (2022) 399:473–86. doi: 10.1016/S0140-6736(21)01730-X 35093231

[2] Solmi M Seitidis G Mavridis D Correll CU Dragioti E Guimond S . Incidence, prevalence, and global burden of schizophrenia - data, with critical appraisal, from the Global Burden of Disease (GBD) 2019. Mol Psychiatry. (2023) 28:5319–27. doi: 10.1038/s41380-023-02138-4 37500825

[3] Leucht S Priller J Davis JM . Antipsychotic drugs: a concise review of history, classification, indications, mechanism, efficacy, side effects, dosing, and clinical application. Am J Psychiatry. (2024) 181:865–78. doi: 10.1176/appi.ajp.20240738 39350614

[4] McCutcheon RA Krystal JH Howes OD . Dopamine and glutamate in schizophrenia: biology, symptoms and treatment. World Psychiatry. (2020) 19:15–33. doi: 10.1002/wps.20693 31922684 PMC6953551

[5] Newcomer JW . Second-generation (atypical) antipsychotics and metabolic effects: a comprehensive literature review. CNS Drugs. (2005) 19:1–93. doi: 10.2165/00023210-200519001-00001 15998156

[6] Mitchell AJ Vancampfort D Sweers K van Winkel R Yu W De Hert M . Prevalence of metabolic syndrome and metabolic abnormalities in schizophrenia and related disorders--a systematic review and meta-analysis. Schizophr Bull. (2013) 39:306–18. doi: 10.1093/schbul/sbr148 22207632 PMC3576174

[7] Grajales D Ferreira V Valverde ÁM . Second-generation antipsychotics and dysregulation of glucose metabolism: beyond weight gain. Cells. (2019) 8:1336. doi: 10.3390/cells8111336 31671770 PMC6912706

[8] Hagi K Nosaka T Dickinson D Lindenmayer JP Lee J Friedman J . Association between cardiovascular risk factors and cognitive impairment in people with schizophrenia: a systematic review and meta-analysis. JAMA Psychiatry. (2021) 78:510–8. doi: 10.1001/jamapsychiatry.2021.0015 33656533 PMC7931134

[9] Vancampfort D Wampers M Mitchell AJ Correll CU De Herdt A Probst M . A meta-analysis of cardio-metabolic abnormalities in drug naïve, first-episode and multi-episode patients with schizophrenia versus general population controls. World Psychiatry. (2013) 12:240–50. doi: 10.1002/wps.20069 24096790 PMC3799255

[10] de Nijs J Schnack HG Koevoets MGJC Kubota M Kahn RS van Haren NEM . Reward-related brain structures are smaller in patients with schizophrenia and comorbid metabolic syndrome. Acta Psychiatr Scand. (2018) 138:581–90. doi: 10.1111/acps.12955 30264457

[11] Zhou J Guo X Liu X Luo Y Chang X He H . Intrinsic therapeutic link between recuperative cerebellar connectivity and psychiatry symptom in schizophrenia patients with comorbidity of metabolic syndrome. Life (Basel). (2023) 13:144. doi: 10.3390/life13010144 36676092 PMC9863013

[12] Chen X Wu X Zhang W Liao K Yu R Lui S . Alterations in regional homogeneity in schizophrenia patients comorbid with metabolic syndrome treated with risperidone or clozapine. J Psychiatr Res. (2025) 181:245–52. doi: 10.1016/j.jpsychires.2024.11.068 39637715

[13] Boyer L Testart J Michel P Richieri R Faget-Agius C Vanoye V . Neurophysiological correlates of metabolic syndrome and cognitive impairment in schizophrenia: a structural equation modeling approach. Psychoneuroendocrinology. (2014) 50:95–105. doi: 10.1016/j.psyneuen.2014.07.019 25199983

[14] Bullmore E Sporns O . Complex brain networks: graph theoretical analysis of structural and functional systems. Nat Rev Neurosci. (2009) 10:186–98. doi: 10.1038/nrn2575 19190637

[15] Sporns O . Structure and function of complex brain networks. Dialogues Clin Neurosci. (2013) 15:247–62. doi: 10.31887/DCNS.2013.15.3/osporns 24174898 PMC3811098

[16] Wu X Chen X Liao K Yu R Chen Y Li K . Characterization of the white matter networks in schizophrenia patients with metabolic syndrome undergoing risperidone or clozapine treatment. Front Neurosci. (2025) 19:1579810. doi: 10.3389/fnins.2025.1579810 40242455 PMC12000066

[17] Zalesky A Cocchi L Fornito A Murray MM Bullmore E . Connectivity differences in brain networks. Neuroimage. (2012) 60:1055–62. doi: 10.1016/j.neuroimage.2012.01.068 22273567

[18] Zalesky A Fornito A Bullmore ET . Network-based statistic: identifying differences in brain networks. Neuroimage. (2010) 53:1197–207. doi: 10.1016/j.neuroimage.2010.06.041 20600983

[19] Cetin-Karayumak S Di Biase MA Chunga N Reid B Somes N Lyall AE . White matter abnormalities across the lifespan of schizophrenia: a harmonized multi-site diffusion MRI study. Mol Psychiatry. (2020) 25:3208–19. doi: 10.1038/s41380-019-0509-y 31511636 PMC7147982

[20] Gao J Tang H Wang Z Li Y Luo N Song M . Graph neural networks and multimodal DTI features for schizophrenia classification: insights from brain network analysis and gene expression. Neurosci Bull. (2025) 41(6):933–50. doi: 10.1007/s12264-025-01385-5 40100543 PMC12158903

[21] First MB Wakefield JC . Defining ‘mental disorder’ in DSM-V. A commentary on: ‘What is a mental/psychiatric disorder? From DSM-IV to DSM-V’ by Stein et al. (2010). Psychol Med. (2010) 40:1779–82. doi: 10.1017/S0033291709992339 20102663

[22] Singh SP Cooper JE Fisher HL Tarrant CJ Lloyd T Banjo J . Determining the chronology and components of psychosis onset: the Nottingham Onset Schedule (NOS). Schizophr Res. (2005) 80:117–30. doi: 10.1016/j.schres.2005.04.018 15978778

[23] Lee AMH Ng CG Koh OH Gill JS Aziz SA . Metabolic syndrome in first episode schizophrenia, based on the National Mental Health Registry of Schizophrenia (NMHR) in a general hospital in Malaysia: a 10-year retrospective cohort study. Int J Environ Res Public Health. (2018) 15:933. doi: 10.3390/ijerph15050933 29735938 PMC5981972

[24] Kang H . Sample size determination and power analysis using the G*Power software. J Educ Eval Health Prof. (2021) 18:17. doi: 10.3352/jeehp.2021.18.17 34325496 PMC8441096

[25] Gardner DM Murphy AL O’Donnell H Centorrino F Baldessarini RJ . International consensus study of antipsychotic dosing. AJP. (2010) 167:686–93. doi: 10.1176/appi.ajp.2009.09060802 20360319

[26] Kay SR Fiszbein A Opler LA . The positive and negative syndrome scale (PANSS) for schizophrenia. Schizophr Bull. (1987) 13:261–76. doi: 10.1093/schbul/13.2.261 3616518

[27] Keefe RSE Goldberg TE Harvey PD Gold JM Poe MP Coughenour L . The Brief Assessment of Cognition in Schizophrenia: reliability, sensitivity, and comparison with a standard neurocognitive battery. Schizophr Res. (2004) 68:283–97. doi: 10.1016/j.schres.2003.09.011 15099610

[28] Simpson GM Angus JW . A rating scale for extrapyramidal side effects. Acta Psychiatr Scand Suppl. (1970) 212:11–9. doi: 10.1111/j.1600-0447.1970.tb02066.x 4917967

[29] Korgaonkar MS Fornito A Williams LM Grieve SM . Abnormal structural networks characterize major depressive disorder: a connectome analysis. Biol Psychiatry. (2014) 76:567–74. doi: 10.1016/j.biopsych.2014.02.018 24690111

[30] Zhang J Wang J Wu Q Kuang W Huang X He Y . Disrupted brain connectivity networks in drug-naive, first-episode major depressive disorder. Biol Psychiatry. (2011) 70:334–42. doi: 10.1016/j.biopsych.2011.05.018 21791259

[31] Sacchet MD Ho TC Connolly CG Tymofiyeva O Lewinn KZ Han LK . Large-scale hypoconnectivity between resting-state functional networks in unmedicated adolescent major depressive disorder. Neuropsychopharmacology. (2016) 41:2951–60. doi: 10.1038/npp.2016.76 27238621 PMC5061890

[32] Baron RM Kenny DA . The moderator-mediator variable distinction in social psychological research: conceptual, strategic, and statistical considerations. J Pers Soc Psychol. (1986) 51:1173–82. doi: 10.1037//0022-3514.51.6.1173 3806354

[33] Kelly S Jahanshad N Zalesky A Kochunov P Agartz I Alloza C . Widespread white matter microstructural differences in schizophrenia across 4322 individuals: results from the ENIGMA schizophrenia DTI working group. Mol Psychiatry. (2018) 23:1261–9. doi: 10.1038/mp.2017.170 29038599 PMC5984078

[34] Klauser P Cropley VL Baumann PS Lv J Steullet P Dwir D . White matter alterations between brain network hubs underlie processing speed impairment in patients with schizophrenia. Schizophr Bull Open. (2021) 2:sgab033. doi: 10.1093/schizbullopen/sgab033 34901867 PMC8650074

[35] Klauser P Baker ST Cropley VL Bousman C Fornito A Cocchi L . White matter disruptions in schizophrenia are spatially widespread and topologically converge on brain network hubs. Schizophr Bull. (2017) 43:425–35. doi: 10.1093/schbul/sbw100 27535082 PMC5605265

[36] Jiang Y Duan M Li X Huang H Zhao G Li X . Function-structure coupling: white matter functional magnetic resonance imaging hyper-activation associates with structural integrity reductions in schizophrenia. Hum Brain Mapp. (2021) 42:4022–34. doi: 10.1002/hbm.25536 34110075 PMC8288085

[37] Pergola G Selvaggi P Trizio S Bertolino A Blasi G . The role of the thalamus in schizophrenia from a neuroimaging perspective. Neurosci Biobehav Rev. (2015) 54:57–75. doi: 10.1016/j.neubiorev.2015.01.013 25616183

[38] Huang AS Wimmer RD Lam NH Wang BA Suresh S Roeske MJ . A prefrontal thalamocortical readout for conflict-related executive dysfunction in schizophrenia. Cell Rep Med. (2024) 5:101802. doi: 10.1016/j.xcrm.2024.101802 39515319 PMC11604477

[39] Menon V Palaniyappan L Supekar K . Integrative brain network and salience models of psychopathology and cognitive dysfunction in schizophrenia. Biol Psychiatry. (2023) 94:108–20. doi: 10.1016/j.biopsych.2022.09.029 36702660

[40] Yan Z Rein B . Mechanisms of synaptic transmission dysregulation in the prefrontal cortex: pathophysiological implications. Mol Psychiatry. (2022) 27:445–65. doi: 10.1038/s41380-021-01092-3 33875802 PMC8523584

[41] Rashid B Dev SI Esterman M Schwarz NF Ferland T Fortenbaugh FC . Aberrant patterns of default‐mode network functional connectivity associated with metabolic syndrome: a resting‐state study. Brain Behav. (2019) 9:e01333. doi: 10.1002/brb3.1333 31568716 PMC6908882

[42] Rashid B Poole VN Fortenbaugh FC Esterman M Milberg WP McGlinchey RE . Association between metabolic syndrome and resting-state functional brain connectivity. Neurobiol Aging. (2021) 104:1–9. doi: 10.1016/j.neurobiolaging.2021.03.012 33951557 PMC8225583

[43] Park B Lee MJ Kim M Kim SH Park H . Structural and functional brain connectivity changes between people with abdominal and non-abdominal obesity and their association with behaviors of eating disorders. Front Neurosci. (2018) 12:741. doi: 10.3389/fnins.2018.00741 30364290 PMC6193119

[44] Baek K Morris LS Kundu P Voon V . Disrupted resting-state brain network properties in obesity: decreased global and putaminal cortico-striatal network efficiency. Psychol Med. (2017) 47:585–96. doi: 10.1017/S0033291716002646 27804899 PMC5426347

[45] Després JP Lemieux I . Abdominal obesity and metabolic syndrome. Nature. (2006) 444:881–7. doi: 10.1038/nature05488 17167477

[46] Neeland IJ Lim S Tchernof A Gastaldelli A Rangaswami J Ndumele CE . Metabolic syndrome. Nat Rev Dis Primers. (2024) 10:77. doi: 10.1038/s41572-024-00563-5 39420195

[47] He H Sui J Yu Q Turner JA Ho BC Sponheim SR . Altered small-world brain networks in schizophrenia patients during working memory performance. PloS One. (2012) 7:e38195. doi: 10.1371/journal.pone.0038195 22701611 PMC3368895

[48] Lynall ME Bassett DS Kerwin R McKenna PJ Kitzbichler M Muller U . Functional connectivity and brain networks in schizophrenia. J Neurosci. (2010) 30:9477–87. doi: 10.1523/JNEUROSCI.0333-10.2010 20631176 PMC2914251

[49] Latora V Marchiori M . Efficient behavior of small-world networks. Phys Rev Lett. (2001) 87:198701. doi: 10.1103/PhysRevLett.87.198701 11690461

[50] Tononi G Edelman GM Sporns O . Complexity and coherency: integrating information in the brain. Trends Cognit Sci. (1998) 2:474–84. doi: 10.1016/s1364-6613(98)01259-5 21227298

[51] Frey BM Petersen M Mayer C Schulz M Cheng B Thomalla G . Characterization of white matter hyperintensities in large-scale MRI-studies. Front Neurol. (2019) 10:238. doi: 10.3389/fneur.2019.00238 30972001 PMC6443932

[52] Petersen M Hoffstaedter F Nägele FL Mayer C Schell M Rimmele DL . A latent clinical-anatomical dimension relating metabolic syndrome to brain structure and cognition. bioRxiv. (2023) 12:RP93246. doi: 10.1101/2023.02.22.529531 38512127 PMC10957178

[53] Szczepanski SM Knight RT . Insights into human behavior from lesions to the prefrontal cortex. Neuron. (2014) 83:1002–18. doi: 10.1016/j.neuron.2014.08.011 25175878 PMC4156912

[54] Zhou Y Fan L Qiu C Jiang T . Prefrontal cortex and the dysconnectivity hypothesis of schizophrenia. Neurosci Bull. (2015) 31:207–19. doi: 10.1007/s12264-014-1502-8 25761914 PMC5563697

[55] Lowe CJ Reichelt AC Hall PA . The prefrontal cortex and obesity: a health neuroscience perspective. Trends Cognit Sci. (2019) 23:349–61. doi: 10.1016/j.tics.2019.01.005 30824229

[56] Vainik U García-García I Dagher A . Uncontrolled eating: a unifying heritable trait linked with obesity, overeating, personality and the brain. Eur J Neurosci. (2019) 50:2430–45. doi: 10.1111/ejn.14352 30667547

[57] Takaeko Y Maruhashi T Kajikawa M Kishimoto S Yamaji T Harada T . Lower triglyceride levels are associated with better endothelial function. J Clin Lipidol. (2021) 15:500–11. doi: 10.1016/j.jacl.2021.04.004 34006457

[58] Yamada S Takahashi S Keeser D Keller-Varady K Schneider-Axmann T Raabe FJ . Impact of excessive abdominal obesity on brain microstructural abnormality in schizophrenia. Psychiatry Res Neuroimaging. (2024) 344:111878. doi: 10.1016/j.pscychresns.2024.111878 39226869

[59] Jackson RL Bajada CJ Rice GE Cloutman LL Lambon Ralph MA . An emergent functional parcellation of the temporal cortex. Neuroimage. (2018) 170:385–99. doi: 10.1016/j.neuroimage.2017.04.024 28419851

[60] van Meer F van der Laan LN Adan RAH Viergever MA Smeets PAM . What you see is what you eat: an ALE meta-analysis of the neural correlates of food viewing in children and adolescents. Neuroimage. (2015) 104:35–43. doi: 10.1016/j.neuroimage.2014.09.069 25285373

[61] Bhaya-Grossman I Chang EF . Speech computations of the human superior temporal gyrus. Annu Rev Psychol. (2022) 73:79–102. doi: 10.1146/annurev-psych-022321-035256 34672685 PMC9447996

[62] Zhang P Wu GW Yu FX Liu Y Li MY Wang Z . Abnormal regional neural activity and reorganized neural network in obesity: evidence from resting-state fMRI. Obes (Silver Spring). (2020) 28:1283–91. doi: 10.1002/oby.22839 32510870

[63] Bini J Parikh L Lacadie C Hwang JJ Shah S Rosenberg SB . Stress-level glucocorticoids increase fasting hunger and decrease cerebral blood flow in regions regulating eating. NeuroImage Clin. (2022) 36:103202. doi: 10.1016/j.nicl.2022.103202 36126514 PMC9486604

[64] Stevenson RJ Boutelle K . Hunger, satiety, and their vulnerabilities. Nutrients. (2024) 16:3013. doi: 10.3390/nu16173013 39275328 PMC11397003

