# Supplementary material for: Disruption of prefrontal and superior temporal gyrus white matter networks in antipsychotic-treated schizophrenia with comorbid metabolic syndrome
Source: Front Psychiatry. 2026 Aug 28;17:1878935. doi: 10.3389/fpsyt.2026.1878935 (PMC13573662; doi:10.3389/fpsyt.2026.1878935)
Supplement: Supplementary file 1 [file Table1.docx]

**Supplementary Table S1. White matter network connections with differences among SZ-MS, SZ-nMS, and HC groups**

| **Region 1** | **Abbreviation of Region 1** | **Region 2** | **Abbreviation of Region 2** | ***F* value** |
| --- | --- | --- | --- | --- |
| **SZ-MS group vs. HC group** |  |  |  |  |
| Left precental gyrus | PreCG.L | Right superior frontal gyrus, dorsolateral | SFGdor.R | 4.129 |
| Right superior frontal gyrus, dorsolateral | SFGdor.R | Left supplementary motor area | SMA.L | 4.263 |
| Left superior frontal gyrus, dorsolateral | SFGdor.L | Right supplementary motor area | SMA.R | 3.243 |
| Left supplementary motor area | SMA.L | Right supplementary motor area | SMA.R | 3.544 |
| Right superior frontal gyrus, dorsolateral | SFGdor.R | Left superior frontal gyrus, medial | SFGmed.L | 3.169 |
| Left postcentral gyrus | PoCG.L | Right postcentral gyrus | PoCG.R | 3.159 |
| Right postcentral gyrus | PoCG.R | Left superior parietal gyrus | SPG.L | 3.615 |
| Left superior frontal gyrus, medial | SFGmed.L | Left precuneus | PCUN.L | 3.520 |
| Right median cingulate and paracingulate gyri | DCG.R | Left precuneus | PCUN.L | 4.287 |
| Right postcentral gyrus | PoCG.R | Left precuneus | PCUN.L | 4.362 |
| Right superior frontal gyrus, dorsolateral | SFGdor.R | Right thalamus | THA.R | 3.864 |
| Left precental gyrus | PreCG.L | Left middle temporal gyrus | MTG.L | 4.410 |
| Left supramarginal gyrus | SMG.L | Left middle temporal gyrus | MTG.L | 3.174 |
| Right thalamus | THA.R | Right temporal pole: middle temporal gyrus | TPOmid.R | 3.193 |
| Left precental gyrus | PreCG.L | Left inferior temporal gyrus | ITG.L | 3.214 |
| **SZ-nMS group vs. HC group** |  |  |  |  |
| Right postcentral gyrus | PoCG.R | Left superior parietal gyrus | SPG.L | 3.431 |
| Left inferior frontal gyrus, triangular part | IFGtriang.L | Left thalamus | THA.L | 2.622 |
| Left hippocampus | HIP.L | Left thalamus | THA.L | 3.022 |
| Left superior parietal gyrus | SPG.L | Left thalamus | THA.L | 2.733 |
| Left hippocampus | HIP.L | Left temporal pole: superior temporal gyrus | TPOsup.L | 3.296 |
| **SZ-MS group vs. SZ-nMS group** |  |  |  |  |
| Left superior frontal gyrus, dorsolateral | SFGdor.L | Left inferior frontal gyrus, triangular part | IFGtriang.L | 3.709 |
| Left superior frontal gyrus, dorsolateral | SFGdor.L | Left inferior frontal gyrus, orbital part | ORBinf.L | 2.680 |
| Left inferior frontal gyrus, triangular part | IFGtriang.L | Left supplementary motor area | SMA.L | 2.820 |
| Left supplementary motor area | SMA.L | Left superior frontal gyrus, medial | SFGmed.L | 2.888 |

These white matter network connections formed a connected network identified by a network-based statistics approach (p < 0.05, corrected). See Figure 1 for the graphical presentation of these connections.

**Supplementary Table S2.** **Comparison of global topological properties among the SZ-MS, SZ-nMS, and HC groups.**

| **Metric** | **SZ-MS group** | **SZ-nMS group** | **HC group** | ***F* value** | ***p* value** | **Post hoc analysis** | | |
| --- | --- | --- | --- | --- | --- | --- | --- | --- |
|  |  |  |  |  |  | **SZ-MS vs. SZ-nMS** | **SZ-MS vs. HC** | **SZ-nMS vs. HC** |
| Cp | 0.135±0.006 | 0.138±0.008 | 0.137±0.005 | 1.682 | 0.194 | 0.084 | 0.211 | 0.614 |
| Gamma | 1.126±0.196 | 1.026±0.115 | 0.939±0.084 | 9.776 | ＜0.001 | **0.035*** | **＜0.001***** | **0.043*** |
| Lambda | 0.336±0.010 | 0.330±0.006 | 0.327±0.006 | 8.037 | 0.001 | **0.028*** | **＜0.001***** | 0.122 |
| Lp | 0.680±0.050 | 0.651±0.029 | 0.631±0.022 | 10.492 | ＜0.001 | **0.017*** | **＜0.001***** | 0.061 |
| Sigma | 0.999±0.143 | 0.929±0.087 | 0.860±0.065 | 9.756 | ＜0.001 | **0.045*** | **＜0.001***** | **0.033*** |
| Eg | 0.132±0.008 | 0.138±0.006 | 0.142±0.005 | 11.556 | ＜0.001 | **0.017*** | **＜0.001***** | **0.037*** |
| Eloc | 0.200±0.010 | 0.204±0.008 | 0.206±0.004 | 3.851 | 0.026 | **0.046*** | **0.012*** | 0.638 |

*Significant group difference at *p* < 0.05, ****p* < 0.001.

Note: Cp, clustering coefficient; Gamma, normalized clustering coefficient; Lambda, normalized characteristic path length; Lp, characteristic path length; Sigma, small-worldness; Eg, global efficiency; Eloc, local efficiency.

**Supplementary Table S3.** **Comparison of nodal degree centrality (DC) among the SZ-MS, SZ-nMS, and HC groups.**

| **Brain region** | **SZ-MS group** | **SZ-nMS group** | **HC group** | ***F* value** | ***p* value** | **Post hoc analysis (Bonferroni-corrected)** | | |
| --- | --- | --- | --- | --- | --- | --- | --- | --- |
|  |  |  |  |  |  | **SZ-MS vs. SZ-nMS** | **SZ-MS vs. HC** | **SZ-nMS vs. HC** |
| Frontal_Sup_R | 4.065±1.198 | 4.997±1.032 | 5.383±0.909 | 8.736 | ＜0.001 | **0.004**** | **＜0.001***** | 0.404 |
| Frontal_Sup_Medial_L | 3.247±0.611 | 3.885±0.746 | 4.338±0.963 | 10.829 | ＜0.001 | **0.019*** | **＜0.001***** | **0.047*** |
| Thalamus_L | 2.776±1.319 | 2.916±1.200 | 4.653±1.352 | 13.749 | ＜0.001 | 0.630 | **＜0.001***** | **＜0.001***** |
| Thalamus_R | 2.154±1.013 | 2.275±0.847 | 3.329±1.050 | 9.878 | ＜0.001 | 0.829 | **＜0.001***** | **0.001**** |

*Significant group difference at *p* < 0.05, ***p* < 0.01, ****p* < 0.001.

Note: Frontal_Sup_R, the right dorsolateral superior frontal gyrus; Frontal_Sup_Medial_L, the left medial superior frontal gyrus; Thalamus_L, the left thalamus; Thalamus_R, the right thalamus.

**Supplementary Table S4.** **Comparison of nodal efficiency (NE) among the SZ-MS, SZ-nMS, and HC groups.**

| **Brain region** | **SZ-MS group** | **SZ-nMS group** | **HC group** | ***F* value** | ***p* value** | **Post hoc analysis (Bonferroni-corrected)** | | |
| --- | --- | --- | --- | --- | --- | --- | --- | --- |
|  |  |  |  |  |  | **SZ-MS vs. SZ-nMS** | **SZ-MS vs. HC** | **SZ-nMS vs. HC** |
| Frontal_Sup_R | 0.148±0.015 | 0.160±0.011 | 0.165±0.009 | 10.867 | ＜0.001 | **0.002**** | **＜0.001***** | 0.294 |
| Frontal_Inf_Tri_L | 0.130±0.015 | 0.144±0.014 | 0.150±0.011 | 12.027 | ＜0.001 | **0.002**** | **＜0.001***** | 0.199 |
| Frontal_Inf_Orb_L | 0.131±0.012 | 0.143±0.015 | 0.144±0.012 | 7.483 | ＜0.001 | **0.004**** | **＜0.001***** | 0.707 |
| Frontal_Sup_Medial_L | 0.140±0.008 | 0.149±0.008 | 0.155±0.012 | 13.133 | ＜0.001 | **0.011*** | **＜0.001***** | **0.027*** |
| Calcarine_R | 0.139±0.013 | 0.145±0.015 | 0.154±0.010 | 9.138 | ＜0.001 | 0.127 | **＜0.001***** | **0.015*** |
| Postcentral_L | 0.136±0.011 | 0.145±0.010 | 0.149±0.010 | 8.563 | ＜0.001 | **0.009**** | **＜0.001***** | 0.244 |
| Parietal_Sup_R | 0.138±0.017 | 0.152±0.015 | 0.157±0.011 | 9.446 | ＜0.001 | **0.010**** | **＜0.001***** | 0.154 |
| SupraMarginal_L | 0.110±0.008 | 0.113±0.006 | 0.119±0.006 | 8.975 | ＜0.001 | 0.168 | **＜0.001***** | **0.012*** |
| SupraMarginal_R | 0.122±0.010 | 0.126±0.012 | 0.133±0.006 | 9.392 | ＜0.001 | 0.253 | **＜0.001***** | **0.005**** |
| Precuneus_L | 0.154±0.013 | 0.162±0.017 | 0.170±0.010 | 8.772 | ＜0.001 | 0.082 | **＜0.001***** | **0.030*** |
| Thalamus_L | 0.138±0.018 | 0.143±0.016 | 0.161±0.012 | 12.305 | ＜0.001 | 0.301 | **＜0.001***** | **0.001**** |
| Thalamus_R | 0.133±0.016 | 0.138±0.012 | 0.151±0.012 | 10.240 | ＜0.001 | 0.264 | **＜0.001***** | **0.003**** |
| Temporal_Sup_R | 0.127±0.012 | 0.135±0.009 | 0.143±0.012 | 11.260 | ＜0.001 | **0.017*** | **＜0.001***** | **0.041*** |

*Significant group difference at *p* < 0.05, ***p* < 0.01, ****p* < 0.001.

Note: Frontal_Sup_R, the right dorsolateral superior frontal gyrus; Frontal_Inf_Tri_L, the triangular part of the inferior frontal gyrus; Frontal_Inf_Orb_L, the left orbital inferior frontal gyrus; Frontal_Sup_Medial_L, the left medial superior frontal gyrus; Calcarine_R, the right calcarine fissure and surrounding cortex; Postcentral_L, the left postcentral gyrus; Parietal_Sup_R, the right superior parietal gyrus; SupraMarginal_L, the left supramarginal gyrus; SupraMarginal_R, the right supramarginal gyrus; Precuneus_L, the left precuneus; Thalamus_L, the left thalamus; Thalamus_R, the right thalamus; Temporal_Sup_R, the right superior temporal gyrus.

**Supplementary Table S5. Comparison of nodal shortest path length (NLP) among the SZ-MS, SZ-nMS, and HC groups.**

| **Brain region** | **SZ-MS group** | **SZ-nMS group** | **HC group** | ***F* value** | ***p* value** | **Post hoc analysis (Bonferroni-corrected)** | | |
| --- | --- | --- | --- | --- | --- | --- | --- | --- |
|  |  |  |  |  |  | **SZ-MS vs. SZ-nMS** | **SZ-MS vs. HC** | **SZ-nMS vs. HC** |
| Frontal_Sup_R | 0.611±0.070 | 0.561±0.039 | 0.544±0.032 | 9.799 | ＜0.001 | **0.002**** | **＜0.001***** | 0.390 |
| Frontal_Inf_Tri_L | 0.695±0.078 | 0.628±0.060 | 0.602±0.044 | 13.535 | ＜0.001 | **0.001**** | **＜0.001***** | 0.192 |
| Frontal_Inf_Orb_L | 0.691±0.066 | 0.632±0.070 | 0.625±0.054 | 7.206 | ＜0.001 | **0.005**** | **＜0.001***** | 0.638 |
| Frontal_Sup_Medial_L | 0.644±0.040 | 0.604±0.033 | 0.583±0.046 | 13.081 | ＜0.001 | **0.006**** | **＜0.001***** | **0.047*** |
| Calcarine_R | 0.651±0.064 | 0.625±0.067 | 0.585±0.040 | 9.039 | ＜0.001 | 0.142 | **＜0.001***** | **0.014*** |
| Postcentral_L | 0.664±0.059 | 0.622±0.045 | 0.604±0.041 | 8.817 | ＜0.001 | **0.007**** | **＜0.001***** | 0.265 |
| Parietal_Sup_R | 0.659±0091 | 0.596±0.060 | 0.574±0.046 | 9.163 | ＜0.001 | **0.009**** | **＜0.001***** | 0.188 |
| SupraMarginal_R | 0.742±0.070 | 0.717±0.071 | 0.674±0.036 | 9.183 | ＜0.001 | 0.296 | **＜0.001***** | **0.005**** |
| Precuneus_L | 0.587±0.055 | 0.560±0.060 | 0.530±0.032 | 8.806 | ＜0.001 | 0.099 | **＜0.001***** | **0.024*** |
| Thalamus_L | 0.660±0.097 | 0.634±0.080 | 0.559±0.043 | 10.020 | ＜0.001 | 0.295 | **＜0.001***** | **0.003**** |
| Thalamus_R | 0.685±0.083 | 0.654±0.057 | 0.599±0.056 | 9.886 | ＜0.001 | 0.183 | **＜0.001***** | **0.006**** |
| Temporal_Sup_R | 0.71±0.074 | 0.667±0.047 | 0.633±0.055 | 10.665 | ＜0.001 | **0.012*** | **＜0.001***** | 0.078 |

*Significant group difference at *p* < 0.05, ***p* < 0.01, ****p* < 0.001.

Note: Frontal_Sup_R, the right dorsolateral superior frontal gyrus; Frontal_Inf_Tri_L, the triangular part of the inferior frontal gyrus; Frontal_Inf_Orb_L, the left orbital inferior frontal gyrus; Frontal_Sup_Medial_L, the left medial superior frontal gyrus; Calcarine_R, the right calcarine fissure and surrounding cortex; Postcentral_L, the left postcentral gyrus; Parietal_Sup_R, the right superior parietal gyrus; SupraMarginal_R, the right supramarginal gyrus; Precuneus_L, the left precuneus; Thalamus_L, the left thalamus; Thalamus_R, the right thalamus; Temporal_Sup_R, the right superior temporal gyrus.
